# Supplementary material for: Functional protein dynamics in a crystal
Source: Nat Commun. 2024 Apr 15;15:3244. doi: 10.1038/s41467-024-47473-4 (PMC11018856; doi:10.1038/s41467-024-47473-4)
Supplement: Supplementary file 3 — Description of Additional Supplementary Files [file 41467_2024_47473_MOESM3_ESM.pdf]

Description of Additional Supplementary Files

File Name: Supplementary Movie 1
Description: Dynamics of the central unit cell (containing four PDZ domains) isolated from the 3x3x3 supercell simulated with the Amber ff14SB force field (replica 1). Chains with the same orientation are shown with the same color. The last 1 microsecond (from t=6.0 to 7.0 microseconds) of the simulation is shown, with one second of the video corresponding to 25 ns of simulation.

File Name: Supplementary Movie 2
Description: Dynamics of the central unit cell (containing four PDZ domains) isolated from the 3x3x3 supercell simulated with the CHARMM36m force field (replica 1). Chains with the same orientation are shown with the same color. The last 1 microsecond (from t=9.0 to 10.0 microsecond) of the simulation is shown, with one second of the video corresponding to 25 ns of simulation.

File Name: Supplementary Movie 3
Description: Dynamics of a single PDZ domain in solution (without a ligand) simulated with the Amber ff14SB force field (replica 1). The last 1 microsecond (from t=2.0 to 3.0 microsecond) of the simulation is shown, with one second of the video corresponding to 25 ns of simulation.

File Name: Supplementary Movie 4
Description: Dynamics of a single PDZ domain in solution (with a ligand shown in gray) simulated with the Amber ff14SB force field (replica 1). The last 1 microsecond (from t=1.0 to 2.0 microseconds) of the simulation is shown, with one second of the video corresponding to 25 ns of simulation.

File Name: Supplementary Movie 5
Description: Visualization of the lattice dynamics for replica 2 of the 3x3x3 supercell (with 108 copies) simulated using the Amber ff14SB force field. The instantaneous centers-of-mass of all 108 protein chains were projected onto the ac crystallographic plane via inverse crystallographic transformations (Supplementary Note 7). Pre-production (60 ns indicated with t) and production (the first 1 microsecond, indicated with t_p) simulations are shown. In this animation, the two clusters of points (visible in the NPT simulation, i.e. after t=20 ns of pre-production) represent chains shifted relative to each other in two different orientations (exhibiting melting symmetry, as described in Supplementary Note 8).

File Name: Supplementary Movie 6
Description: Visualization of the lattice dynamics for replica 2 of the 3x3x3 supercell (with 108 copies) simulated using the CHARMM36m force field. The instantaneous centers-of-mass of all 108 protein chains were projected onto the ac crystallographic plane via inverse crystallographic transformations (Supplementary Note 7). Pre-production (60 ns indicated with t) and production (the first 1 microsecond, indicated with t_p) simulations are shown. In this animation, the two clusters of points (visible in the NPT simulation, i.e. after t=20 ns of pre-production) represent chains shifted relative to each other in two different orientations (exhibiting melting symmetry, as described in Supplementary Note 8).
