# Supplementary material for: Functional protein dynamics in a crystal
Source: Nat Commun. 2024 Apr 15;15:3244. doi: 10.1038/s41467-024-47473-4 (PMC11018856; doi:10.1038/s41467-024-47473-4)
Supplement: Supplementary file 10 — Reporting Summary [file 41467_2024_47473_MOESM10_ESM.pdf]

nature portfolio

Corresponding author(s): Sarah Rauscher

Last updated by author(s): Mar 22, 2024

Reporting Summary

Nature Portfolio wishes to improve the reproducibility of the work that we publish. This form provides structure for consistency and transparency in reporting. For further information on Nature Portfolio policies, see our Editorial Policies and the Editorial Policy Checklist.

Statistics

For all statistical analyses, confirm that the following items are present in the figure legend, table legend, main text, or Methods section.

n/a	Confirmed	
☐	☒	The exact sample size (*n*) for each experimental group/condition, given as a discrete number and unit of measurement
☐	☒	A statement on whether measurements were taken from distinct samples or whether the same sample was measured repeatedly
☒	☐	The statistical test(s) used AND whether they are one- or two-sided<br>*Only common tests should be described solely by name; describe more complex techniques in the Methods section.*
☒	☐	A description of all covariates tested
☒	☐	A description of any assumptions or corrections, such as tests of normality and adjustment for multiple comparisons
☐	☒	A full description of the statistical parameters including central tendency (e.g. means) or other basic estimates (e.g. regression coefficient) AND variation (e.g. standard deviation) or associated estimates of uncertainty (e.g. confidence intervals)
☒	☐	For null hypothesis testing, the test statistic (e.g. *F*, *t*, *r*) with confidence intervals, effect sizes, degrees of freedom and *P* value noted<br>*Give P values as exact values whenever suitable.*
☒	☐	For Bayesian analysis, information on the choice of priors and Markov chain Monte Carlo settings
☒	☐	For hierarchical and complex designs, identification of the appropriate level for tests and full reporting of outcomes
☒	☐	Estimates of effect sizes (e.g. Cohen's *d*, Pearson's *r*), indicating how they were calculated

Our web collection on statistics for biologists contains articles on many of the points above.

Software and code

Policy information about availability of computer code

Data collection	Molecular dynamics trajectories were generated using GROMACS 2019.1
Data analysis	The initial structure for MD simulations was prepared with CHARMM-GUI web server (https://www.charmm-gui.org/) and PROPKA web-interface (https://server.poissonboltzmann.org/). MD simulation data was analyzed in Python (version 3.7) using modules MDAnalysis (version 2.0.0), scikit-learn (version 1.2.0), umap-learn (version 0.4.6), and MSMBuilder (version 3.8.0). Analysis of the crystallographic water sites was performed using the LAWS algorithm (https://github.com/rauscher-lab/LAWS). Crystal structures were aligned using PyMOL (version 2.5.5). The molecular structures were visualized using VMD (version 1.9.4) and UCSF ChimeraX (version 1.6.1).

For manuscripts utilizing custom algorithms or software that are central to the research but not yet described in published literature, software must be made available to editors and reviewers. We strongly encourage code deposition in a community repository (e.g. GitHub). See the Nature Portfolio guidelines for submitting code & software for further information.

Data

Policy information about availability of data

All manuscripts must include a data availability statement. This statement should provide the following information, where applicable:

- Accession codes, unique identifiers, or web links for publicly available datasets
- A description of any restrictions on data availability
- For clinical datasets or third party data, please ensure that the statement adheres to our policy

Full details of computational methods, supplementary results and discussion are provided in Supplementary Information. The MD simulation data (the initial and

final coordinates) for all systems have been deposited to the Zenodo repository and are available from https://doi.org/10.5281/zenodo.7987473. The data underlying Figs. 1-5 are available in the Source Data file. The crystallographic data used in this study are available from the Protein Data Bank under accession code 5E11 [https://doi.org/10.2210/pdb5E11/pdb] (Ground state of the PDZ2 domain) and 5E21 [https://doi.org/10.2210/pdb5E21/pdb] (PDZ2 domain in electric field). The Top8000 dataset used in this study for assessing dihedral angle distributions in the crystal environment is available from GitHub [https://github.com/rlabduke/reference_data].

Human research participants

Policy information about studies involving human research participants and Sex and Gender in Research.

Reporting on sex and gender	n/a
Population characteristics	n/a
Recruitment	n/a
Ethics oversight	n/a

Note that full information on the approval of the study protocol must also be provided in the manuscript.

Field-specific reporting

Please select the one below that is the best fit for your research. If you are not sure, read the appropriate sections before making your selection.

- [x] Life sciences
- [ ] Behavioural & social sciences
- [ ] Ecological, evolutionary & environmental sciences

For a reference copy of the document with all sections, see nature.com/documents/nr-reporting-summary-flat.pdf

Life sciences study design

All studies must disclose on these points even when the disclosure is negative.

Sample size	In the study, the analysis was conducted on the entire simulated dataset. The 'n' referenced in the manuscript refers to the number of replicas, the number of chains in the supercell, or the number of residues in the protein chain. The meaning of 'n' is specified in each instance.
Data exclusions	No data were excluded from analysis.
Replication	Three simulation replicas were run for each crystal simulation and 10 simulation replicas were run for each solution system. Results from the individual replica simulations are consistent with each other. Results from all replicate simulations are reported in the paper and discussed.
Randomization	All simulation replicas were initialized with randomly seeded initial velocities to provide various initial conditions.
Blinding	Blinding was not applicable to the study because the simulations and analysis were performed using computer algorithms, not involving human subjects or any subjective interpretations.

Reporting for specific materials, systems and methods

We require information from authors about some types of materials, experimental systems and methods used in many studies. Here, indicate whether each material, system or method listed is relevant to your study. If you are not sure if a list item applies to your research, read the appropriate section before selecting a response.

Materials & experimental systems

n/a	Involved in the study	
x		Antibodies
x		Eukaryotic cell lines
x		Palaeontology and archaeology
x		Animals and other organisms
x		Clinical data
x		Dual use research of concern

Methods

n/a	Involved in the study	
x		ChIP-seq
x		Flow cytometry
x		MRI-based neuroimaging
